# Supplementary material for: Gender Disparities in Epidemiology, Treatment, and Outcome for Head and Neck Cancer in Germany: A Population-Based Long-Term Analysis from 1996 to 2016 of the Thuringian Cancer Registry
Source: Cancers (Basel). 2020 Nov 18;12(11):3418. doi: 10.3390/cancers12113418 (PMC7698743; doi:10.3390/cancers12113418)
Supplement: Supplementary file 1 [file cancers-12-03418-s001.pdf]

cancers

MDPI

Supplementary Materials

Gender disparities in epidemiology, treatment, and outcome for head and neck cancer in Germany: A population-based long-term analysis from 1996 to 2016 of the Thuringian cancer registry

Andreas Dittberner, Benedikt Friedl, Andrea Wittig, Jens Buentzel, H. Kaftan, Daniel Böger, Andreas Müller, Stefan Schultze-Mosgau, Peter Schlattmann, Thomas Ernst, and Orlando Guntinas-Lichius

Supplementary Table S1

Supplementary Table S1. Distribution of head and neck cancer patients according to clinical and demographic parameters.

Parameter	Frequency (N)	%
All	8288	100
Year of diagnosis	362	4.2
1996	362	4.4
1997	340	4.1
1998	334	4.0
1999	361	4.4
2000	393	4.7
2001	415	5.0
2002	348	4.2
2003	447	5.4
2004	482	5.8
2005	423	5.1
2006	438	5.3
2007	447	5.4
2008	470	5.7
2009	428	5.2
2010	471	5.7
2011	478	5.8
2012	322	3.9
2013	313	3.8
2014	334	4.0
2015	333	4.0
2016	349	4.2
Tumor registry region		
Jena	2007	24.2
Erfurt	3226	38.9
Gera	997	12.0
Nordhausen	646	7.8
Suhl	1412	17.0
Patient with residence in Thuringia		
Yes	7502	90.5
No	786	9.5

Gender		
Male	6540	78.9
Female	1748	21.1
	**Mean±SD**	**Median, Range**
Age, years	60.9±11.9	60, 12-100
Follow-up of all patients, months	45.1±45.7	29.1, 0-268,9
Follow-up of patients alive, months	59.6±49.6	50.4, 0-268.9

Supplementary Table S2

Supplementary Table S2. T classification in relation to N classification in head neck cancer patients*.

	N0	N1	N2	N3	all
T1	1439	168	278	21	1906
T2	917	230	570	62	1779
T3	496	160	569	38	1263
T4	484	224	1015	161	1884
Sum	3336	782	2432	282	6832

* AJCC 2017 Staging was not clearly specified in some patients: Tx = 1189 and Nx = 1411.

Supplementary Table S3

Supplementary Table S3. Stage distribution for subsites of head and neck cancer.*

**Subsite**	**Stage I**	**Stage II**	**Stage III**	**Stage IV**	**all**
Lip	157	27	13	9	206
Cavity of mouth	465	329	256	759	1809
Oropharynx	153	169	266	1366	1954
Nasopharynx	10	12	27	97	146
Hypopharynx	18	28	104	671	821
Larynx	531	252	275	432	1490
Nose and paranasal sinus	19	25	38	82	164
Salivary gland	82	69	72	130	353
Not specified	0	0	1	0	1
Sum	1435	911	1052	3546	6944

*Because T or N were not clearly specified in some cases, stage grouping was not possible for all 8288 patients. Be aware that T and N classification rules and stage grouping guidelines differ for the different subsites.

Supplementary Table S4

**Supplementary Table S4**. Distribution of distant metastases in 362 M+ patients at primary diagnosis for the different subsites of head and neck cancer.											
**Subsite**	**PUL**	**LYM**	**HEP**	**OSS**	**OTH**	**BRA**	**SKI**	**PLE**	**ADR**	**Total number of metastases**	**Number of patients**
Cavity of mouth	31	10	7	12	6	1	4	1	1	73	57
Oropharynx	71	19	13	18	8	3	3	3	2	140	121
Nasopharynx	3	1	5	5	1	2	0	0	0	17	12
Hypopharynx	63	27	9	7	7	5	2	2	2	124	95
Larynx	30	4	7	3	5	0	1	0	0	50	43
Nose and paranasal sinus	3	0	4	0	1	0	0	0	0	8	10
Salivary gland	12	5	7	4	2	5	1	0	1	37	24
Sum	213	66	52	49	30	16	11	6	6	449	362

Pulmonary PUL; Osseous OSS; Hepatic HEP; Brain BRA; Lymph nodes LYM;; Pleura PLE; Adrenal ADR; Skin SKI; Other OTH. No distant metastases found in Bone marrow MAR, genitourinary tract GEN, and Peritoneum PER. No distant metastases detected in patients with lip cancer.

Supplementary Table S5

**Supplementary Table S5**. Relation of tumor stage to therapy strategy.				
**Stage**	**No therapy**	**Monotherapy**	**Multimodal therapy**	**Sum**
**AJCC**				
I	31	1075	329	1435
II	20	389	502	911
III	19	232	801	1052
IV	202	667	2677	3546
Unstaged	164	472	380	1016
All	436	2835	4689	7960
**SEER**				
Localized	85	1701	1508	3294
Regionalized	133	580	2563	3276
Distant	51	77	232	360
Unstaged	167	477	386	1030
All	436	2835	4689	7960

Stage grouping was not possible for all 8288 patients. AJCC = American Joint Committee on Cancer; SEER = Surveillance, Epidemiology, and End Results Program

Supplementary Table S6

Supplementary Table S6. Crude incidence of head and neck cancer for the different age cohorts from 1996 to 2016.											
**Parameter**	**1996**	**1997**	**1998**	**1999**	**2000**	**2001**	**2002**	**2003**	**2004**	**2005**	**2006**	**2007**
**All, years**												
All ages	13.43	13.10	13.06	13.45	15.14	16.07	13.22	16.99	18.48	16.24	16.51	17.16
10-14	0.00	0.00	0.00	0.00	0.00	0.00	0.00	0.00	0.00	0.00	0.00	1.43
15-19	0.00	0.00	0.57	0.57	0.57	1.17	0.00	0.00	0.00	0.00	0.00	0.79
20-24	0.74	0.00	0.00	0.69	0.66	0.00	0.00	0.00	0.00	0.00	0.00	0.66
25-29	1.77	0.61	1.89	0.00	0.71	1.52	0.79	0.00	0.00	0.73	0.00	0.00
30-34	0.49	0.51	0.00	0.55	1.14	1.18	0.00	0.65	2.10	2.25	1.61	0.00
35-39	7.86	4.83	2.40	2.86	5.30	4.93	3.07	5.34	4.47	1.75	4.86	1.91
40-44	9.65	10.19	12.79	5.12	11.70	9.58	5.97	16.71	7.33	12.83	7.57	8.89
45-49	16.44	17.14	14.45	14.39	20.36	19.24	11.48	25.74	20.50	15.16	19.67	21.96
50-54	23.98	25.38	15.15	29.86	27.45	20.26	25.98	23.37	28.99	33.80	28.84	30.54
55-59	27.33	35.03	30.24	32.85	38.64	25.41	23.52	30.41	41.76	26.02	31.61	43.52
60-64	32.51	29.01	41.18	32.38	32.55	44.57	34.35	34.84	45.94	32.39	39.61	26.80
65-69	36.15	32.42	28.82	31.22	27.97	42.50	28.35	32.71	39.57	34.64	32.92	29.53
70-74	28.25	25.18	24.78	32.36	25.22	30.25	30.24	41.43	31.39	31.23	32.33	21.20
75-79	20.98	15.86	20.14	24.88	31.62	34.16	24.63	34.35	32.12	24.76	20.17	41.36
80-84	16.91	9.82	23.73	14.34	18.57	35.93	30.92	19.04	29.20	30.22	17.43	21.94
≥85	21.41	22.98	22.43	21.68	26.36	17.48	21.31	17.17	37.85	21.72	29.51	18.17
**Male, years**												
All ages	22.33	23.04	21.30	22.33	24.66	26.66	22.12	28.00	30.10	27.11	27.10	27.38
10-14	0.00	0.00	0.00	0.00	0.00	0.00	0.00	0.00	0.00	0.00	0.00	0.00
15-19	0.00	0.00	1.10	0.00	1.11	1.13	0.00	0.00	0.00	0.00	0.00	0.00
20-24	1.38	0.00	0.00	0.00	0.00	0.00	0.00	0.00	0.00	0.00	0.00	1.23
25-29	1.11	1.14	0.00	0.00	1.31	2.78	1.43	0.00	0.00	1.34	0.00	0.00
30-34	0.95	0.98	0.00	1.04	1.08	1.12	0.00	1.22	2.59	2.76	2.95	0.00
35-39	13.41	6.59	4.68	4.64	10.28	8.60	3.95	8.22	7.51	2.23	9.26	2.41
40-44	15.91	18.95	20.04	9.01	21.87	15.74	11.65	27.79	10.47	22.07	12.73	13.16
45-49	26.26	25.74	26.13	27.17	32.80	30.62	18.44	43.31	31.95	26.62	26.36	34.98
50-54	43.76	47.75	25.73	42.65	44.38	32.30	45.28	38.20	50.72	59.31	51.64	51.84
55-59	48.39	62.76	49.50	58.79	63.35	48.73	41.48	53.58	76.14	49.20	59.17	71.90
60-64	55.57	54.19	71.96	65.23	58.50	83.64	64.50	59.39	82.90	53.55	68.11	48.66
65-69	68.87	60.90	44.99	58.51	45.96	74.64	53.79	59.09	71.47	62.58	57.84	48.02
70-74	64.86	59.63	52.21	53.45	47.96	63.39	57.51	72.08	51.98	55.59	54.49	37.13
75-79	34.22	43.08	53.44	46.57	68.38	59.79	50.43	70.77	65.46	46.61	33.41	70.39
80-84	23.00	26.98	67.67	31.50	38.52	64.96	55.87	50.15	41.23	57.05	38.68	47.43
≥85	54.94	53.08	41.63	51.28	52.00	44.32	24.13	26.42	67.06	49.15	54.83	40.10
**Female, years**												
All ages	5.10	3.74	5.28	5.03	6.08	5.95	4.71	6.43	7.30	5.76	6.29	7.28
10-14	0.00	0.00	0.00	0.00	0.00	0.00	0.00	0.00	0.00	0.00	0.00	2.92
15-19	0.00	0.00	0.00	1.18	0.00	1.22	0.00	0.00	0.00	0.00	0.00	1.62
20-24	0.00	0.00	0.00	1.50	1.43	0.00	0.00	0.00	0.00	0.00	0.00	0.00
25-29	2.51	0.00	4.10	0.00	0.00	0.00	0.00	0.00	0.00	0.00	0.00	0.00
30-34	0.00	0.00	0.00	0.00	1.20	1.25	0.00	0.00	1.52	1.65	0.00	0.00
35-39	2.01	2.98	0.00	0.98	0.00	1.02	2.12	2.22	1.17	1.22	0.00	1.35
40-44	3.11	1.04	5.23	1.05	1.04	3.10	0.00	5.04	4.02	3.05	2.08	4.32
45-49	6.21	8.17	2.27	1.09	7.45	7.42	4.25	7.50	8.58	3.20	12.70	8.36

50-54	4.35	2.99	4.55	16.93	10.21	7.94	6.13	8.10	6.66	7.61	5.41	8.69
55-59	7.08	8.33	11.67	7.79	14.72	2.78	5.99	7.72	7.83	2.97	4.04	14.97
60-64	11.62	6.15	13.12	2.26	8.63	8.38	6.32	11.95	11.40	12.55	12.81	6.13
65-69	11.10	9.91	15.87	8.76	12.87	15.17	6.58	9.95	11.76	10.10	10.88	13.12
70-74	8.93	6.03	9.01	19.40	10.41	7.50	10.69	18.79	15.77	12.33	14.84	8.47
75-79	15.13	3.81	5.44	15.34	15.24	22.36	12.17	15.61	13.75	11.95	12.02	22.67
80-84	14.59	3.38	7.25	7.89	10.99	24.83	21.38	7.17	24.63	19.85	8.89	11.15
≥85	10.61	13.45	16.38	12.60	18.68	9.67	20.51	14.62	29.75	13.94	22.19	11.74
	**2008**	**2009**	**2010**	**2011**	**2012**	**2013**	**2014**	**2015**	**2016**	**RR***	**95%CI upper**	**95%CI lower**
**All, years**												
All ages	17.87	16.51	17.27	19.26	16.00	15.34	16.72	16.61	17.09			
10-14	0.00	0.00	0.00	0.00	1.22	0.00	0.00	0.00	0.00	1.96	0.60	6.44
15-19	0.00	0.00	0.00	0.00	1.44	0.00	0.00	0.00	0.00	0.77	0.38	1.57
20-24	2.67	0.68	0.00	0.00	0.00	0.94	2.14	0.00	1.25	1.47	0.90	2.41
25-29	1.40	0.70	0.00	0.00	0.73	0.73	1.47	0.00	0.76	0.80	0.56	1.16
30-34	1.68	0.00	0.00	0.79	0.77	0.00	0.00	0.00	1.48	0.97	0.69	1.36
35-39	1.35	2.17	5.44	0.00	1.77	2.65	0.86	3.26	1.56	0.75	0.64	0.88
40-44	9.29	6.31	9.57	7.79	6.08	3.55	6.77	8.77	3.38	0.87	0.79	0.96
45-49	23.18	15.55	21.25	21.36	11.06	12.69	13.29	16.33	12.99	0.97	0.91	1.04
50-54	31.30	29.17	31.70	26.08	24.65	27.49	20.51	21.73	23.82	1.00	0.95	1.06
55-59	29.36	36.14	39.35	42.06	33.77	32.29	27.75	28.70	42.88	1.04	0.99	1.09
60-64	42.92	31.93	30.91	39.67	34.72	33.41	42.80	36.18	31.98	1.01	0.96	1.05
65-69	38.76	31.82	28.10	40.80	32.05	27.72	27.13	31.39	32.89	0.99	0.93	1.04
70-74	25.08	31.84	28.86	31.86	24.88	17.73	28.86	28.69	27.36	0.97	0.92	1.03
75-79	31.05	23.36	25.56	36.23	23.09	25.93	27.41	20.51	21.90	1.00	0.93	1.07
80-84	12.19	22.09	15.71	9.89	16.94	19.93	30.89	24.12	22.76	1.01	0.91	1.12
≥85	15.06	14.45	3.94	20.96	23.73	14.01	11.74	22.63	14.17	0.89	0.80	1.00
**Male, years**												
All ages	30.26	27.24	28.71	32.03	25.41	24.63	25.34	26.78	26.26			
10-14	0.00	0.00	0.00	0.00	2.38	0.00	0.00	0.00	0.00	3.13	0.34	28.48
15-19	0.00	0.00	0.00	0.00	0.00	0.00	0.00	0.00	0.00	0.37	0.08	1.75
20-24	3.76	1.28	0.00	0.00	0.00	1.80	2.06	0.00	0.00	1.53	0.81	2.88
25-29	2.57	1.30	0.00	0.00	0.00	1.37	0.00	0.00	0.00	0.75	0.45	1.24
30-34	3.10	0.00	0.00	1.47	1.43	0.00	0.00	0.00	1.38	0.95	0.65	1.40
35-39	2.53	1.35	5.77	0.00	3.32	3.31	1.61	4.59	0.00	0.67	0.56	0.82
40-44	15.85	11.05	14.94	12.52	10.34	4.04	8.51	16.47	6.34	0.85	0.77	0.94
45-49	38.44	23.52	37.52	37.86	14.14	18.12	18.89	24.41	20.06	0.95	0.89	1.02
50-54	52.21	50.09	56.08	40.89	38.23	41.94	34.41	36.75	39.62	0.99	0.93	1.05
55-59	49.13	64.19	64.17	73.09	56.45	61.36	39.74	43.93	62.38	1.01	0.96	1.06
60-64	76.41	61.89	58.21	69.29	57.27	59.08	68.86	61.72	56.80	0.99	0.94	1.04
65-69	73.41	54.20	44.89	72.98	53.32	45.50	51.29	55.15	51.36	0.97	0.91	1.03
70-74	44.60	52.33	47.87	56.26	39.29	31.72	45.20	52.85	50.39	0.92	0.86	0.99
75-79	58.93	41.69	44.48	58.67	43.90	36.68	45.19	31.70	33.69	0.91	0.83	0.99
80-84	24.48	45.12	29.40	24.18	35.24	27.00	48.37	41.88	32.46	0.94	0.82	1.07
≥85	18.74	8.91	8.38	31.50	65.90	33.91	18.85	46.83	27.69	0.88	0.75	1.04
**Female, years**												
All ages	5.87	6.12	6.18	6.99	6.94	6.39	8.41	6.74	8.16			
10-14	0.00	0.00	0.00	0.00	0.00	0.00	0.00	0.00	0.00	1.41	0.30	6.54
15-19	0.00	0.00	0.00	0.00	2.97	0.00	0.00	0.00	0.00	1.10	0.47	2.56
20-24	1.43	0.00	0.00	0.00	0.00	0.00	2.22	0.00	2.66	1.38	0.63	3.05

25-29	0.00	0.00	0.00	0.00	1.59	0.00	3.17	0.00	1.64	0.87	0.51	1.48
30-34	0.00	0.00	0.00	0.00	0.00	0.00	0.00	0.00	1.60	1.03	0.51	2.06
35-39	0.00	3.12	5.07	0.00	0.00	1.89	0.00	1.75	3.34	1.07	0.76	1.51
40-44	2.26	1.19	3.74	2.69	1.42	3.00	4.80	0.00	0.00	0.95	0.74	1.23
45-49	7.22	7.19	4.15	4.34	7.87	7.06	7.42	7.77	5.39	1.06	0.92	1.24
50-54	9.84	7.67	6.55	10.97	10.81	12.80	6.36	6.42	7.66	1.06	0.93	1.23
55-59	9.43	7.93	14.44	11.19	11.23	3.39	15.82	13.50	23.39	1.20	1.06	1.35
60-64	11.06	3.21	4.56	11.13	12.89	8.53	17.55	11.48	8.00	1.07	0.95	1.22
65-69	7.89	11.81	13.01	11.91	12.84	11.57	5.03	9.52	15.82	1.00	0.88	1.13
70-74	9.33	15.10	13.17	11.65	12.86	6.03	15.16	8.35	7.89	0.99	0.88	1.12
75-79	12.48	10.75	12.21	20.16	7.97	18.01	14.10	12.02	12.89	1.03	0.91	1.16
80-84	6.64	10.93	8.66	2.18	6.62	15.80	20.30	13.05	16.57	1.00	0.85	1.18
≥85	13.96	16.12	2.58	17.59	9.73	7.08	9.15	13.40	8.80	0.89	0.76	1.04

*RR = relative risk per five year period; CI = 95% confidence interval

Supplementary Table S7

Supplementary Table S7. Crude incidence of head and neck cancer for four different age cohorts from 1996 to 2016.

Parameter	1996	1997	1998	1999	2000	2001	2002	2003	2004	2005	2006	2007
**All, years**												
<50	4.87	4.37	4.30	3.26	5.65	5.32	3.07	7.22	5.29	5.12	5.37	5.71
50-64	27.97	30.37	29.53	31.86	32.91	30.92	28.50	29.48	38.37	30.93	32.61	33.94
65-79	29.33	25.87	25.22	29.92	28.30	36.21	28.04	35.90	34.63	31.15	29.64	29.67
≥80	18.89	16.27	23.06	18.33	21.40	27.19	26.88	17.26	32.30	27.08	22.16	20.40
**Male, years**												
<50	7.76	6.92	6.93	5.58	9.48	8.39	5.01	11.74	7.84	8.53	7.99	8.39
50-64	49.19	55.73	49.70	56.50	55.50	56.02	51.39	49.93	68.56	54.19	58.22	57.85
65-79	59.41	56.93	49.07	54.33	52.00	67.84	54.37	65.65	63.50	57.06	51.64	49.22
≥80	36.12	38.95	55.13	41.50	40.00	56.23	44.24	38.26	49.12	54.55	44.09	44.91
**Female, years**												
<50	1.76	1.61	1.47	0.74	1.49	1.98	0.94	2.26	2.49	1.37	2.47	2.73
50-64	7.74	6.14	10.24	8.22	11.12	6.58	6.16	9.45	8.69	7.95	7.17	10.11
65-79	11.32	6.86	10.47	14.42	12.74	14.70	9.55	14.36	13.06	11.29	12.41	14.11
≥80	12.79	8.42	12.05	10.51	15.15	17.39	21.00	10.12	26.55	17.56	14.37	11.41
	**2008**	**2009**	**2010**	**2011**	**2012**	**2013**	**2014**	**2015**	**2016**			
**All, years**												
<50	6.42	4.31	6.23	5.27	3.57	3.45	3.84	4.42	3.27			
50-64	34.44	32.20	34.28	35.35	30.76	30.72	29.91	28.82	33.01			
65-79	31.26	29.83	27.51	35.96	26.59	22.95	28.14	26.48	27.08			
≥80	14.27	19.77	10.76	14.60	19.90	17.28	21.40	23.45	18.94			
**Male, years**												
<50	10.51	6.46	10.04	8.92	4.87	4.79	4.88	6.95	4.33			
50-64	58.87	58.17	59.57	59.73	50.03	53.24	46.86	47.34	53.05			
65-79	58.23	50.84	45.39	62.64	45.06	36.91	47.11	46.19	44.58			
≥80	25.73	32.95	22.38	26.65	45.92	29.50	37.39	43.73	30.70			
**Female, years**												
<50	1.88	1.94	1.99	1.25	2.14	1.98	2.70	1.60	2.08			
50-64	10.02	6.20	8.89	11.09	11.58	8.32	13.05	10.39	13.03			
65-79	9.56	12.73	12.86	14.12	11.40	11.43	12.39	10.01	12.35			
≥80	9.88	14.46	5.88	9.33	8.10	11.54	13.63	13.22	12.80			

Supplementary Table S8

**Supplementary Table S8.** European standard rate (ESR) incidence of head and neck cancer for the different age cohorts from 1996 to 2016.												
**Parameter**	**1996**	**1997**	**1998**	**1999**	**2000**	**2001**	**2002**	**2003**	**2004**	**2005**	**2006**	**2007**
All	12.25	11.77	11.64	12.08	13.44	13.79	11.28	14.16	15.35	13.03	13.18	13.41
10-14	0.00	0.00	0.00	0.00	0.00	0.00	0.00	0.00	0.00	0.00	0.00	0.08
15-19	0.00	0.00	0.03	0.03	0.04	0.07	0.00	0.00	0.00	0.00	0.00	0.05
20-24	0.05	0.00	0.00	0.05	0.04	0.00	0.00	0.00	0.00	0.00	0.00	0.04
25-29	0.12	0.04	0.13	0.00	0.05	0.11	0.06	0.00	0.00	0.05	0.00	0.00
30-34	0.04	0.04	0.00	0.04	0.08	0.09	0.00	0.05	0.15	0.16	0.12	0.00
35-39	0.57	0.35	0.17	0.21	0.38	0.36	0.22	0.39	0.32	0.13	0.35	0.14
40-44	0.70	0.74	0.93	0.37	0.85	0.70	0.44	1.22	0.53	0.94	0.55	0.65
45-49	1.18	1.24	1.04	1.04	1.47	1.39	0.83	1.86	1.48	1.09	1.42	1.58
50-54	1.66	1.75	1.05	2.06	1.90	1.40	1.79	1.61	2.00	2.33	1.99	2.11
55-59	1.75	2.24	1.94	2.10	2.47	1.63	1.51	1.95	2.67	1.67	2.02	2.79
60-64	1.89	1.68	2.39	1.88	1.89	2.58	1.99	2.02	2.66	1.88	2.30	1.55
65-69	1.68	1.51	1.34	1.46	1.30	1.98	1.32	1.52	1.84	1.61	1.53	1.38
70-74	1.14	1.02	1.00	1.30	1.02	1.22	1.22	1.67	1.27	1.26	1.30	0.85
75-79	0.69	0.52	0.66	0.82	1.04	1.12	0.81	1.13	1.06	0.82	0.66	1.36
80-84	0.40	0.23	0.56	0.34	0.43	0.84	0.72	0.45	0.68	0.71	0.41	0.51
≥85	0.38	0.41	0.40	0.38	0.47	0.31	0.38	0.30	0.67	0.39	0.52	0.32
	**2008**	**2009**	**2010**	**2011**	**2012**	**2013**	**2014**	**2015**	**2016**			
All	13.76	12.32	12.88	13.94	11.58	10.96	11.70	11.75	11.93			
10-14	0.00	0.00	0.00	0.00	0.07	0.00	0.00	0.00	0.00			
15-19	0.00	0.00	0.00	0.00	0.09	0.00	0.00	0.00	0.00			
20-24	0.18	0.05	0.00	0.00	0.00	0.06	0.14	0.00	0.08			
25-29	0.10	0.05	0.00	0.00	0.05	0.05	0.10	0.00	0.05			
30-34	0.12	0.00	0.00	0.06	0.06	0.00	0.00	0.00	0.11			
35-39	0.10	0.16	0.39	0.00	0.13	0.19	0.06	0.24	0.11			
40-44	0.68	0.46	0.70	0.57	0.44	0.26	0.49	0.64	0.25			
45-49	1.67	1.12	1.53	1.54	0.80	0.91	0.96	1.18	0.94			
50-54	2.16	2.01	2.19	1.80	1.70	1.90	1.42	1.50	1.64			
55-59	1.88	2.31	2.52	2.69	2.16	2.07	1.78	1.84	2.74			
60-64	2.49	1.85	1.79	2.30	2.01	1.94	2.48	2.10	1.85			
65-69	1.81	1.48	1.31	1.90	1.49	1.29	1.26	1.46	1.53			
70-74	1.01	1.28	1.16	1.28	1.00	0.71	1.16	1.16	1.10			
75-79	1.02	0.77	0.84	1.19	0.76	0.85	0.90	0.68	0.72			
80-84	0.29	0.52	0.37	0.23	0.40	0.47	0.72	0.56	0.53			
≥85	0.27	0.26	0.07	0.37	0.42	0.25	0.21	0.40	0.25			

Supplementary Table S9

Supplementary Table S9. Crude incidence of head and neck cancer subsites from 1996 to 2016.

**Parameter**	**1996**	**1997**	**1998**	**1999**	**2000**	**2001**	**2002**	**2003**	**2004**	**2005**	**2006**	**2007**
All	12.60	12.39	12.27	12.83	14.55	15.49	12.74	16.45	17.89	15.82	16.70	17.04
Lip	0.44	0.56	0.73	0.77	0.57	0.50	0.62	0.76	0.59	0.26	0.30	0.57
Oral cavity	3.17	2.46	2.87	3.01	4.18	3.84	2.66	4.53	4.31	3.88	4.65	4.65
Oropharynx	3.17	2.14	2.91	3.18	3.36	3.22	3.54	4.03	4.95	4.65	4.86	5.09
Nasopharynx	0.60	0.52	0.28	0.33	0.33	0.41	0.42	0.46	0.30	0.34	0.26	0.22
Hypopharynx	1.28	1.94	1.50	1.38	1.31	1.90	1.17	1.68	2.45	2.22	1.94	2.00
Larynx	3.05	3.31	2.55	3.18	3.65	4.05	3.21	3.99	3.89	3.28	3.06	2.78
Nose/paranasal	0.24	0.52	0.53	0.12	0.25	0.37	0.33	0.17	0.30	0.04	0.56	0.74
Middle ear	0.00	0.00	0.00	0.00	0.00	0.00	0.04	0.00	0.00	0.00	0.00	0.00
Salivary gland	0.64	0.85	0.89	0.81	0.82	0.99	0.67	0.84	1.06	1.15	1.03	1.00
Not classifiable	0.00	0.08	0.00	0.04	0.08	0.21	0.08	0.00	0.04	0.00	0.04	0.00
	**2008**	**2009**	**2010**	**2011**	**2012**	**2013**	**2014**	**2015**	**2016**	**RR***	**95%CI upper**	**95%CI lower**
All	17.95	16.57	18.20	19.15	14.80	14.45	15.47	15.39	16.12	1.07	1.05	1.09
Lip	0.44	0.40	0.58	0.23	0.51	0.18	0.32	0.28	0.46	0.84	0.76	0.94
Oral cavity	5.22	3.50	5.13	4.98	3.77	3.51	3.71	4.16	3.83	1.08	1.04	1.12
Oropharynx	5.09	4.34	5.13	5.44	4.64	4.94	4.26	4.95	4.90	1.16	1.12	1.20
Nasopharynx	0.26	0.35	0.22	0.46	0.37	0.18	0.32	0.32	0.37	0.90	0.79	1.02
Hypopharynx	1.49	2.39	2.10	1.60	1.79	1.85	1.81	1.66	2.08	1.06	1.01	1.12
Larynx	3.51	3.63	2.54	3.70	2.25	2.31	3.84	2.54	3.47	0.97	0.94	1.02
Nose/paranasal	0.70	0.58	0.54	1.19	0.55	0.65	0.51	0.46	0.37	1.22	1.09	1.36
Middle ear	0.00	0.04	0.09	0.05	0.00	0.05	0.05	0.00	0.00	1.85	0.92	3.72
Salivary gland	1.19	1.06	1.12	1.19	0.92	0.78	0.65	1.02	0.65	1.03	0.95	1.11
Not classifiable	0.04	0.27	0.76	0.32	0.00	0.00	0.00	0.00	0.00	1.22	0.95	1.55

*RR = relative risk per five year period, CI = 95% confidence interval

Supplementary Table S10

Supplementary Table S10. Crude incidence of head and neck cancer subsites from 1996 to 2016 for male patients.

**Parameter**	**1996**	**1997**	**1998**	**1999**	**2000**	**2001**	**2002**	**2003**	**2004**	**2005**	**2006**	**2007**
All	20.32	21.13	19.69	20.68	22.96	24.87	20.79	26.92	28.64	26.17	27.09	27.43
Lip	0.74	0.99	1.08	1.25	0.67	0.67	0.93	0.77	0.77	0.35	0.44	0.88
Oral cavity	5.12	4.31	4.65	4.34	6.03	5.56	3.56	6.92	6.11	5.98	7.08	7.50
Oropharynx	5.12	3.31	4.49	5.00	5.45	5.14	5.94	6.58	8.09	7.88	8.04	8.29
Nasopharynx	0.83	0.66	0.50	0.42	0.50	0.67	0.85	0.77	0.43	0.61	0.35	0.26
Hypopharynx	2.40	3.65	2.74	2.42	2.43	3.37	2.12	3.08	4.64	3.64	3.58	3.35
Larynx	5.37	6.30	4.74	6.09	6.37	7.76	6.36	7.43	7.05	5.98	5.59	5.03
Nose/paranasal	0.33	0.75	0.58	0.17	0.25	0.51	0.42	0.17	0.43	0.09	0.61	0.88
Middle ear	0.00	0.00	0.00	0.00	0.00	0.00	0.00	0.00	0.00	0.00	0.00	0.00
Salivary gland	0.41	1.16	0.91	0.92	1.26	1.18	0.59	1.20	1.12	1.65	1.31	1.23
Not classifiable	0.00	0.00	0.00	0.08	0.00	0.00	0.00	0.00	0.00	0.00	0.09	0.00
	**2008**	**2009**	**2010**	**2011**	**2012**	**2013**	**2014**	**2015**	**2016**	**RR***	**95%CI upper**	**95%CI lower**
All	30.35	26.93	28.73	30.76	23.44	23.27	23.32	24.79	24.82	1.06	1.03	1.08
Lip	0.62	0.36	0.72	0.37	0.75	0.19	0.28	0.47	0.47	0.78	0.69	0.90
Oral cavity	8.10	5.21	7.68	7.81	5.23	5.16	4.89	5.80	5.32	1.06	1.01	1.10
Oropharynx	9.26	7.63	9.04	9.11	7.75	8.26	6.40	8.51	7.37	1.17	1.12	1.21
Nasopharynx	0.44	0.36	0.45	0.56	0.56	0.28	0.38	0.65	0.47	0.89	0.77	1.03
Hypopharynx	2.76	4.22	3.61	2.97	3.36	3.38	3.48	2.99	3.83	1.06	1.00	1.13
Larynx	6.59	6.91	4.70	6.69	4.20	3.94	7.05	4.68	6.16	0.97	0.93	1.01
Nose/paranasal	1.07	0.63	0.81	1.67	0.75	0.75	0.56	0.65	0.56	1.24	1.08	1.42
Middle ear	0.00	0.00	0.09	0.00	0.00	0.09	0.00	0.00	0.00	2.60	0.55	12.30
Salivary gland	1.51	1.62	1.63	1.58	0.84	1.22	0.28	1.03	0.65	1.03	0.93	1.14
Not classifiable	0.00	0.00	0.00	0.00	0.00	0.00	0.00	0.00	0.00	0.62	0.17	2.23

*RR = relative risk per five year period, CI = 95% confidence interval

Supplementary Table S11

Supplementary Table S11. Crude incidence of head and neck cancer subsites from 1996 to 2016 for female patients.

Parameter	1996	1997	1998	1999	2000	2001	2002	2003	2004	2005	2006	2007
All	5.31	4.09	5.21	5.33	6.50	6.48	4.99	6.35	7.49	5.79	6.62	6.95
Lip	0.16	0.16	0.39	0.32	0.48	0.32	0.33	0.74	0.42	0.17	0.17	0.26
Oral cavity	1.33	0.71	1.18	1.75	2.41	2.19	1.80	2.23	2.58	1.85	2.29	1.89
Oropharynx	1.33	1.02	1.42	1.43	1.36	1.38	1.23	1.57	1.91	1.51	1.78	1.97
Nasopharynx	0.39	0.39	0.08	0.24	0.16	0.16	0.00	0.16	0.17	0.08	0.17	0.17
Hypopharynx	0.23	0.31	0.32	0.40	0.24	0.49	0.25	0.33	0.33	0.84	0.34	0.69
Larynx	0.86	0.47	0.47	0.40	1.04	0.49	0.16	0.66	0.83	0.67	0.59	0.60
Nose/paranasal	0.16	0.31	0.47	0.08	0.24	0.24	0.25	0.16	0.17	0.00	0.51	0.60
Middle ear	0.00	0.00	0.00	0.00	0.00	0.00	0.08	0.00	0.00	0.00	0.00	0.00
Salivary gland	0.86	0.55	0.87	0.72	0.40	0.81	0.74	0.49	1.00	0.67	0.76	0.77
Not classifiable	0.00	0.16	0.00	0.00	0.16	0.40	0.16	0.00	0.08	0.00	0.00	0.00
	**2008**	**2009**	**2010**	**2011**	**2012**	**2013**	**2014**	**2015**	**2016**	**RR***	**95%CI upper**	**95%CI lower**
All	5.89	6.47	7.93	7.91	6.42	5.91	7.85	6.21	7.59	1.10	1.05	1.15
Lip	0.26	0.44	0.44	0.09	0.27	0.18	0.37	0.09	0.46	0.97	0.80	1.16
Oral cavity	2.43	1.84	2.64	2.25	2.35	1.91	2.56	2.56	2.38	1.14	1.06	1.23
Oropharynx	1.04	1.14	1.32	1.89	1.63	1.73	2.19	1.46	2.47	1.11	1.02	1.21
Nasopharynx	0.09	0.35	0.00	0.36	0.18	0.09	0.27	0.00	0.27	0.90	0.71	1.15
Hypopharynx	0.26	0.61	0.62	0.27	0.27	0.36	0.18	0.37	0.37	1.04	0.88	1.22
Larynx	0.52	0.44	0.44	0.81	0.36	0.73	0.73	0.46	0.82	1.01	0.88	1.15
Nose/paranasal	0.35	0.52	0.26	0.72	0.36	0.55	0.46	0.27	0.18	1.18	0.99	1.42
Middle ear	0.00	0.09	0.09	0.09	0.00	0.00	0.09	0.00	0.00	1.66	0.75	3.66
Salivary gland	0.87	0.52	0.62	0.81	1.00	0.36	1.00	1.00	0.64	1.03	0.91	1.16
Not classifiable	0.09	0.52	1.50	0.63	0.00	0.00	0.00	0.00	0.00	1.26	0.98	1.62

*RR = relative risk per five year period, CI = 95% confidence interval

Supplementary Table S12

Supplementary Table S12. Crude incidence of head and neck cancer for the different stages from 1996 to 2016.

Parameter	1996	1997	1998	1999	2000	2001	2002	2003	2004	2005	2006	2007
**All**												
Stage I	1.97	2.38	2.15	2.00	2.42	3.10	2.75	2.69	3.00	2.52	2.32	2.91
Stage II	1.36	0.97	1.05	1.14	1.52	1.61	1.46	2.14	1.90	1.58	1.98	2.26
Stage III	1.81	1.53	1.66	1.87	1.43	1.90	1.42	1.76	2.11	2.39	1.89	2.09
Stage IV	5.90	5.49	4.86	4.81	5.41	6.11	4.91	6.55	7.74	6.14	7.66	6.61
Unstaged	1.57	2.02	2.55	3.01	3.77	2.77	2.21	3.32	3.13	3.20	2.84	3.17
**Men**												
Stage I	2.81	4.14	3.16	2.67	3.60	4.64	4.50	4.44	4.82	4.16	4.28	4.41
Stage II	2.40	1.49	1.58	1.83	2.35	2.87	2.55	3.42	2.92	2.69	3.23	3.17
Stage III	3.14	2.82	2.82	3.25	2.68	2.95	2.38	2.99	3.61	3.99	3.15	3.62
Stage IV	10.49	10.19	8.14	8.09	9.22	10.88	8.57	11.62	13.76	10.66	12.58	10.67
Unstaged	1.49	2.49	3.99	4.84	5.11	3.54	2.80	4.44	3.53	4.68	3.85	5.56
**Women**												
Stage I	1.17	0.71	1.18	1.35	1.28	1.62	1.06	0.99	1.25	0.92	0.42	1.46
Stage II	0.39	0.47	0.55	0.48	0.72	0.40	0.41	0.91	0.92	0.50	0.76	1.37
Stage III	0.55	0.31	0.55	0.56	0.24	0.89	0.49	0.58	0.67	0.84	0.68	0.60
Stage IV	1.56	1.02	1.74	1.67	1.76	1.54	1.39	1.65	1.91	1.76	2.88	2.66
Unstaged	1.64	1.57	1.18	1.27	2.49	2.02	1.63	2.23	2.75	1.76	1.87	0.86
	**2008**	**2009**	**2010**	**2011**	**2012**	**2013**	**2014**	**2015**	**2016**	**RR***	**95%CI upper**	**95%CI lower**
**All**												
Stage I	3.47	2.61	2.50	3.29	2.30	2.08	3.80	3.28	2.73	1.08	1.03	1.13
Stage II	2.59	1.99	1.87	1.51	1.79	1.48	1.62	1.62	2.08	1.10	1.04	1.17
Stage III	2.19	1.64	1.92	2.65	1.84	1.66	1.81	2.17	2.26	1.06	1.01	1.12
Stage IV	6.37	7.26	7.76	8.68	7.77	7.90	6.99	7.39	7.76	1.12	1.09	1.15
Unstaged	3.34	3.06	4.15	3.02	1.10	1.34	1.25	0.92	1.29	0.91	0.86	0.95
**Men**												
Stage I	5.43	3.86	3.61	5.39	3.55	2.91	5.27	5.14	3.55	1.06	1.01	1.11
Stage II	4.00	3.32	2.89	2.51	2.43	2.06	2.16	2.25	3.17	1.06	1.00	1.14
Stage III	3.92	2.96	3.16	4.46	3.08	2.81	2.73	3.55	3.45	1.04	0.98	1.10
Stage IV	11.92	12.48	14.00	14.59	12.70	13.42	11.38	12.25	12.60	1.10	1.06	1.13
Unstaged	5.07	4.31	5.06	3.81	1.68	2.06	1.79	1.59	2.05	0.93	0.88	0.98
**Women**												
Stage I	1.56	1.40	1.41	1.26	1.09	1.27	2.37	1.46	1.92	1.13	1.03	1.23
Stage II	1.21	0.70	0.88	0.54	1.18	0.91	1.10	1.00	1.01	1.23	1.09	1.39
Stage III	0.52	0.35	0.70	0.90	0.63	0.55	0.91	0.82	1.10	1.16	1.02	1.32
Stage IV	0.95	2.19	1.67	2.97	2.99	2.55	2.74	2.65	3.02	1.21	1.12	1.30
Unstaged	1.65	1.84	3.26	2.25	0.54	0.64	0.73	0.27	0.55	0.86	0.79	0.93

*RR = relative risk per five year period, CI = 95% confidence interval

Supplementary Table S13

**Supplementary Table S13.** Relative proportion* of therapy strategies per year from 1996 to 2016.												
**Parameter**	**1996**	**1997**	**1998**	**1999**	**2000**	**2001**	**2002**	**2003**	**2004**	**2005**	**2006**	**2007**
No therapy**	0.06	0.05	0.08	0.08	0.07	0.04	0.06	0.07	0.05	0.05	0.03	0.04
Chemotherapy alone	0.01	0.02	0.02	0.00	0.01	0.01	0.01	0.02	0.02	0.00	0.01	0.00
Radiotherapy alone	0.12	0.09	0.10	0.10	0.13	0.08	0.10	0.07	0.06	0.07	0.05	0.04
Radiochemotherapy/radio-immunotherapy	0.12	0.13	0.11	0.12	0.13	0.12	0.13	0.15	0.12	0.13	0.10	0.11
Surgery alone	0.25	0.30	0.26	0.22	0.26	0.29	0.27	0.26	0.28	0.28	0.26	0.30
Surgery and chemotherapy/biologicals	0.01	0.01	0.02	0.03	0.00	0.02	0.01	0.02	0.02	0.02	0.02	0.01
Surgery and radiotherapy	0.24	0.24	0.23	0.25	0.22	0.22	0.19	0.21	0.19	0.21	0.24	0.27
Surgery and radiochemotherapy/radio-immunotherapy	0.18	0.17	0.19	0.21	0.19	0.23	0.23	0.20	0.25	0.22	0.29	0.24
Monotherapy	0.38	0.41	0.37	0.32	0.40	0.37	0.38	0.35	0.36	0.36	0.31	0.34
Multimodal therapy	0.55	0.55	0.55	0.61	0.54	0.59	0.56	0.58	0.59	0.59	0.65	0.62
Surgery part of concept	0.69	0.71	0.70	0.70	0.67	0.75	0.71	0.69	0.75	0.74	0.81	0.81
Radiotherapy part of concept	0.67	0.63	0.63	0.68	0.66	0.65	0.65	0.63	0.64	0.64	0.68	0.65
Chemotherapy/biological part of concept	0.29	0.29	0.27	0.29	0.22	0.27	0.31	0.32	0.35	0.34	0.41	0.35
Platin***	0.27	0.26	0.25	0.25	0.21	0.24	0.29	0.27	0.32	0.31	0.39	0.32
5-Fluorouracil	0.04	0.05	0.10	0.05	0.01	0.01	0.02	0.02	0.03	0.00	0.06	0.04
Taxane****	0.00	0.01	0.02	0.01	0.01	0.02	0.01	0.03	0.03	0.01	0.05	0.02
Cetuximab	0.00	0.00	0.00	0.00	0.00	0.00	0.00	0.00	0.01	0.00	0.05	0.06
Monochemotherapy	0.23	0.19	0.12	0.18	0.16	0.20	0.23	0.23	0.26	0.30	0.30	0.28
Polychemotherapy	0.06	0.10	0.15	0.10	0.07	0.08	0.08	0.10	0.10	0.04	0.11	0.07
	**2008**	**2009**	**2010**	**2011**	**2012**	**2013**	**2014**	**2015**	**2016**	**RR****	**95%CI upper**	**95%CI lower**
No therapy***	0.02	0.04	0.06	0.04	0.03	0.07	0.07	0.09	0.08	1.04	0.96	1.12
Chemotherapy alone	0.00	0.01	0.01	0.01	0.01	0.00	0.02	0.01	0.01	0.96	0.80	1.16
Radiotherapy alone	0.05	0.07	0.06	0.06	0.04	0.05	0.05	0.06	0.06	0.83	0.78	0.89
Radiochemotherapy/radio-immunotherapy	0.09	0.09	0.11	0.10	0.16	0.18	0.11	0.13	0.20	1.08	1.02	1.13
Surgery alone	0.29	0.28	0.23	0.31	0.32	0.22	0.34	0.27	0.29	1.07	1.03	1.10
Surgery and chemotherapy/biologicals	0.01	0.01	0.00	0.00	0.01	0.00	0.00	0.01	0.00	0.74	0.62	0.89
Surgery and radiotherapy	0.30	0.23	0.23	0.24	0.14	0.18	0.19	0.18	0.14	0.98	0.94	1.02
Surgery and radiochemotherapy/radio-immunotherapy	0.23	0.28	0.29	0.20	0.19	0.21	0.17	0.21	0.19	1.05	1.01	1.09
Monotherapy	0.34	0.35	0.31	0.38	0.37	0.27	0.42	0.35	0.36	1.00	0.96	1.04
Multimodal therapy	0.64	0.61	0.64	0.58	0.60	0.66	0.52	0.56	0.56	1.33	1.26	1.40
Surgery part of concept	0.83	0.80	0.76	0.79	0.72	0.67	0.73	0.69	0.64	1.04	1.02	1.06
Radiotherapy part of concept	0.68	0.67	0.69	0.63	0.63	0.70	0.56	0.62	0.62	1.03	1.00	1.05
Chemotherapy/biological part of concept	0.33	0.38	0.42	0.35	0.47	0.48	0.35	0.38	0.43	1.16	1.12	1.19
Platin****	0.29	0.30	0.37	0.30	0.41	0.42	0.29	0.36	0.40	1.15	1.11	1.19

5-Fluorouracil	0.06	0.09	0.09	0.17	0.30	0.26	0.16	0.19	0.19	1.85	1.72	1.98
Taxane*****	0.03	0.04	0.05	0.02	0.03	0.02	0.02	0.01	0.00	1.10	0.98	1.25
Cetuximab	0.06	0.15	0.09	0.09	0.11	0.10	0.07	0.05	0.03	2.06	1.85	2.29
Monochemotherapy	0.23	0.24	0.29	0.13	0.09	0.14	0.11	0.17	0.22	1.00	0.96	1.04
Polychemotherapy	0.10	0.13	0.13	0.18	0.27	0.25	0.16	0.18	0.18	1.33	1.26	1.40

*Sum per year = 1; **RR = relative risk per five year period; CI = 95% confidence interval; ***beyond best supportive care; ****Cisplatin or carboplatin; *****paclitaxel or docetaxel

Supplementary Table S14

**Supplementary Table S14.** Distribution of recurrence/tumor progress in the population.		
**Parameter**	**Frequency (N)**	**%**
All	8288	100
Any recurrence		
Yes	1330	16.0
No	6958	84.0
Locoregional recurrence		
Yes	921	11.1
No	7367	88.9
Distant recurrence (rM+)		
Yes	621	7.5
No	7667	92.5
Distant recurrence subsite (rM+)		
All*	814	100
PUL	333	40.9
OSS	116	14.3
LYM	91	11.2
HEP	70	8.6
OTH	73	9.0
SKI	53	6.5
BRA	32	3.9
PLE	30	3.7
ADR	9	1.1
PER	7	0.9
Tumor progress		
Yes	681	8.2
No	7607	91.8
Death		
Yes	4481	54.1
No	3807	45.9
	**Mean/Median**	**95% CI**
Mean recurrence-free survival time, months**	192.9	187.8-198.0
Median recurrence-free survival time, months**	NA	NA
Mean overall survival time, months**	91.3	88.3-94.2
Median overall survival time, months**	54.3	51.8-58.7

*number of distant metastases higher than number of patients with M+

**due to Kaplan-Meier calculation; CI = 95% upper and lower confidence interval

Supplementary Table S15

Supplementary Table S15. Association of baseline and tumor characteristics and on overall survival.

Parameter	Dichotomized	2-year OS %	5-year OS %	log Rank p
All		65.4	48.5	
Gender	Male	63.4	46.2	**<0.0001**
	Female	73.4	57.7	
Age (median: 60 years)	<Median	68.3	52.2	**<0.0001**
	>Median	62.5	44.7	
Site	Lip	85.2	69.7	**<0.0001**
	Oral cavity	64.4	46.8	
	Oropharynx	62.2	46.3	
	Nasopharynx	67.5	49.9	
	Hypopharynx	47.1	30.1	
	Larynx	75.3	56.8	
	Nose/paranasal	66.1	48.9	
	Middle ear	33.0	0	
	Salivary gland	72.9	59.3	
	Not classifiable	0	0	
T classification	T1	87.8	69.5	**<0.0001**
	T2	76.8	54.8	
	T3	67.1	41.8	
	T4	46.4	26.6	
T classification	T1/T2	82.6	62.5	**<0.0001**
	T3/T4	54.7	32.7	
N classification	N0	82.5	61.7	**<0.0001**
	N1	68.2	45.3	
	N2	53.3	35.9	
	N3	31.7	19.3	
N classification	N0	79.8	61.7	**<0.0001**
	N+	57.9	36.7	
M classification	M0	71.4	50.8	**<0.0001**
	M+	26.4	9.9	

UICC stage	I	91.0	74.2	**<0.0001**
	II	78.5	59.2	
	III	76.1	52.7	
	IV	50.3	33.9	
UICC stage	I-II	86.8	67.0	**<0.0001**
	III-IV	59.5	37.9	
Histology	SCC	64.2	46.9	**<0.0001**
	Other	72.1	57.1	
Surgery*	No	40.1	25.6	**<0.0001**
	Yes	77.6	56.4	
Radiotherapy*	No	69.8	55.5	**<0.0001**
	Yes	63.1	44.7	
Chemotherapy/immunotherapy*	No	69.3	53.8	**<0.0001**
	Yes	58.9	38.4	
Therapy	Best supportive care	31.8	23.2	**<0.0001**
	Monotherapy	69.7	55.1	
	Multimodal therapy	65.7	46.7	
Years of treatment	1996-2000	65.3	47.6	0.114
	2001-2005	65.2	47.6	
	2006-2010	67.5	50.0	
	2011-2016	64.8	49.2	

*as part of therapy regime; SCC = Squamous cell carcinoma

Supplementary Table S16

**Supplementary Table S16.** Crude 5-year overall survival rates* in % from 1996 to 2016.				
**Parameter**	**1996-2000**	**2001-2005**	**2006-2010**	**2011-2016**
**All**	47.6	47.6	50.0	49.2
Lip	73.4	65.3	70.6	63.4
Oral cavity	42.7	42.7	51.2	50.2
Oropharynx	42.0	46.9	47.7	49.2
Nasopharynx	55.4	42.6	44.2	54.2
Hypopharynx	27.6	29.7	32.2	30.8
Larynx	58.7	56.3	57.9	53.0
Nose/paranasal	42.8	44.9	53.2	51.4
Middle ear	NA	NA	NA	0
Salivary gland	60.7	63.9	55.3	57.6
Not classifiable	0	NA	NA	NA
**All**				
Stage I	74.8	69.7	78.8	72.3
Stage II	62.8	56.5	56.8	64.2
Stage III	50.0	54.0	54.7	54.4
Stage IV	30.0	31.3	36.9	36.1
**Men**				
Stage I	75.9	69.9	78.5	72.0
Stage II	58.9	54.4	54.3	66.0
Stage III	47.8	51.3	52.0	54.2
Stage IV	28.0	29.2	35.2	34.0
**Women**				
Stage I	71.7	69.1	79.9	72.1
Stage II	75.3	65.6	65.3	59.7
Stage III	62.9	66.0	68.9	54.5
Stage IV	40.5	44.1	48.9	45.0

*related to tumor stage excluding unstaged cases

Supplemental Figures

Figure S1: Relative amount of patients regarding treatment (no therapy, monotherapy, multimodal therapy.

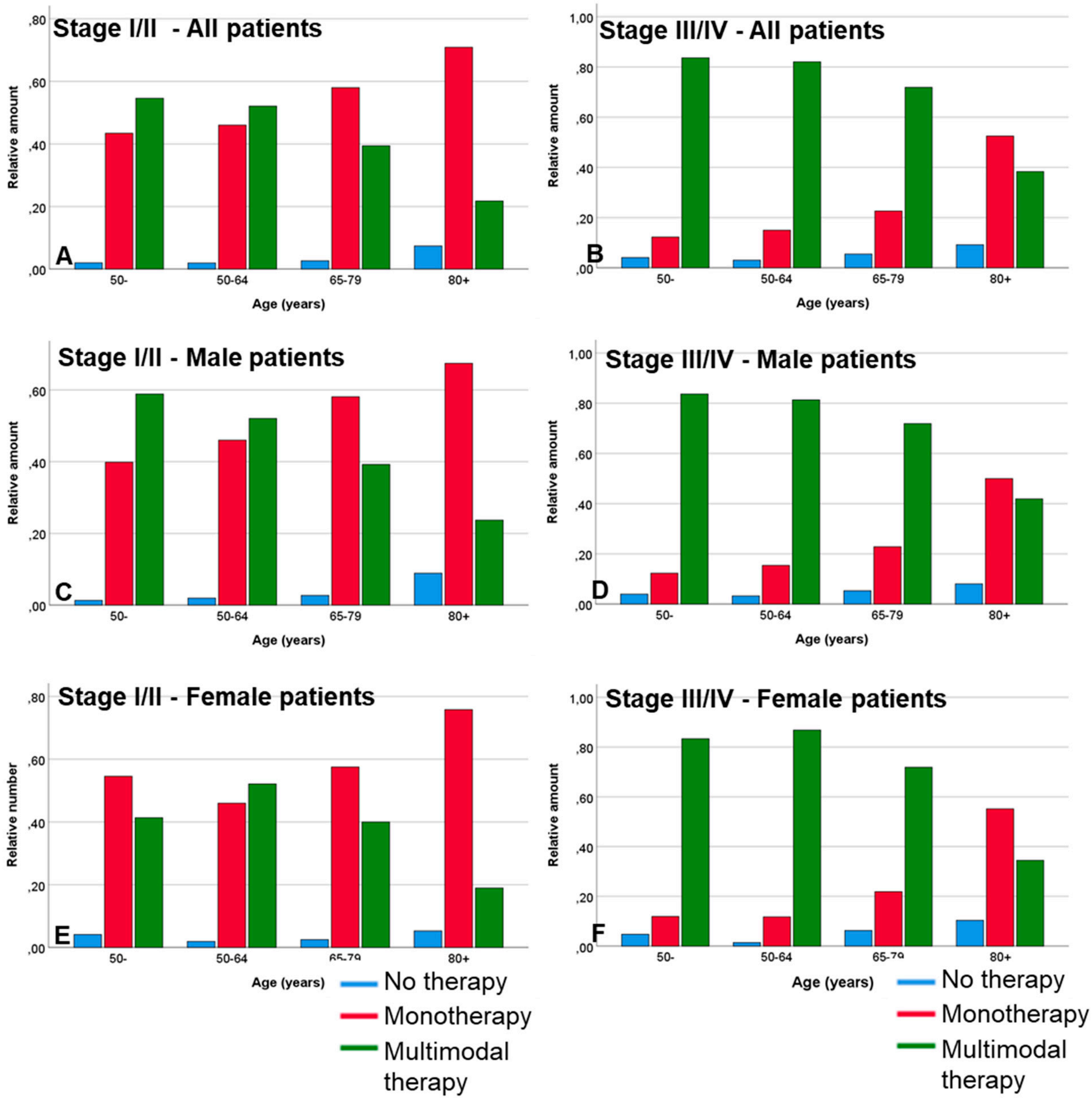

(**A,C,E**) Stage I/II. (**B,D,F**) Stage III/IV. (**A,B**) All patients. (**C,**D) Male patients. (**E,F**) Female patients.

Figure S2: Mean age in years over the years from 1996 to 2016.

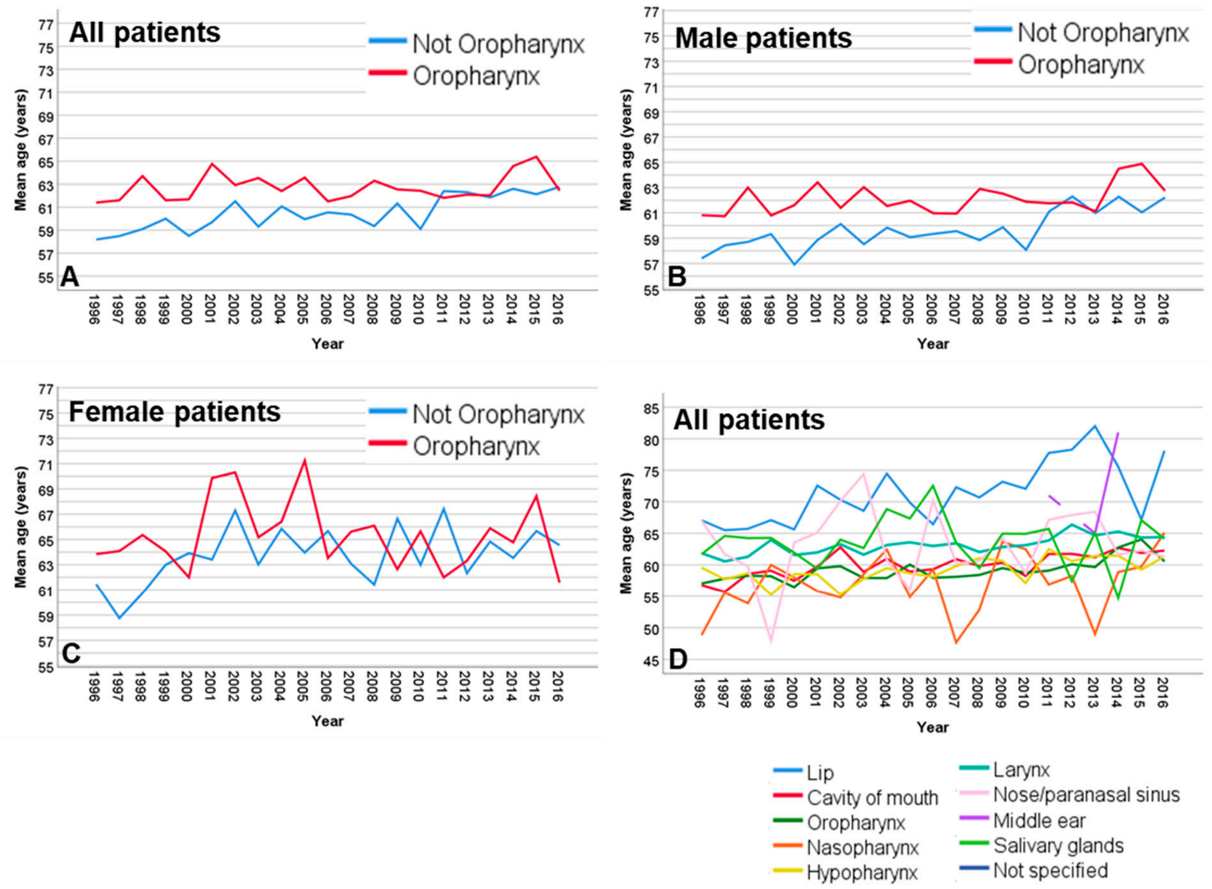

(**A**,**B**,**C**) Oropharyngeal cancer versus non-oropharyngeal cancer. (**A**) All patients. (**B**) Male patients. (**C**) Female patients. (**D**) All tumor subsites of all patients.

Figure S3: 5-year overall survival rates in percentage in relation to the year of primary diagnosis in 5-year periods from 1996 to 2016.

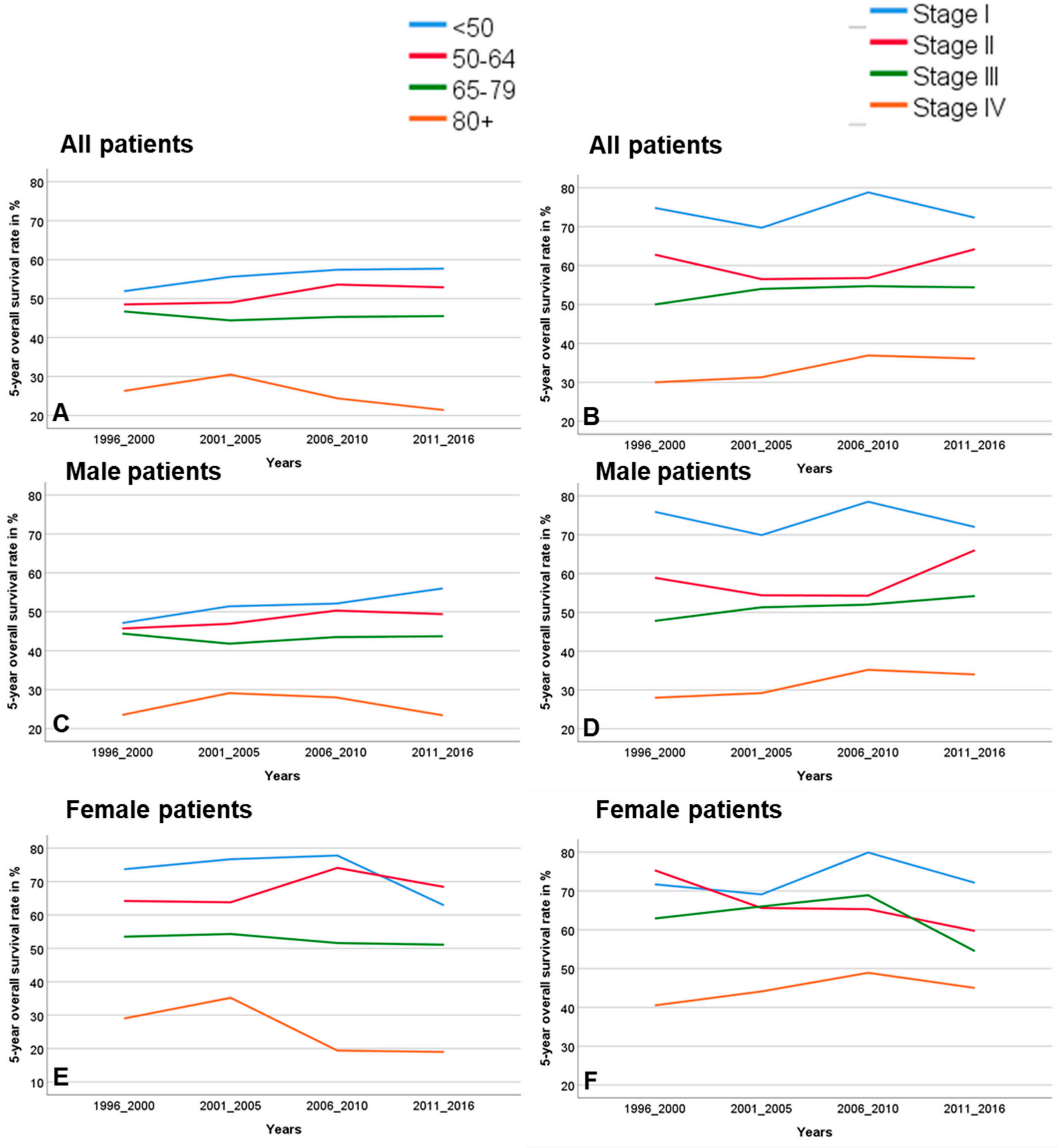

(**A,C,E**) For different age cohorts. (**B,D,F**) For different stages. (**A,B**) All .patients. (**C,D**) Male patients. (**E,F**) Female patients.
